# A novel fatty acid analogue triggers CD36–GPR120 interaction and exerts anti-inflammatory action in endotoxemia

**DOI:** 10.1007/s00018-024-05207-1

**Published:** 2024-04-10

**Authors:** Pierre-Marie Boutanquoi, Amira Sayed Khan, Lidia Cabeza, Lucas Jantzen, Thomas Gautier, Semen Yesylevskyy, Christophe Ramseyer, David Masson, Vincent Van Waes, Aziz Hichami, Naim Akhtar Khan

**Affiliations:** 1 Physiologie de la Nutrition & Toxicologie, UMR U1231 INSERM/Université de Bourgogne/Agro-Sup, Université Bourgogne Franche-Comté, 6 Boulevard Gabriel, 21000 Dijon, France; 2 FCS Bourgogne-Franche Comté, LipSTIC LabEx, Dijon, France; 3 Laboratoire de Recherches Intégratives en Neurosciences et Psychologie Cognitive-UR LINC, UFC, Besançon, France; 4 LIPNESS, UMR U1231 INSERM/UB/Agro-Sup, Université Bourgogne Franche-Comté, 21000 Dijon, France; 5 Institute of Organic Chemistry and Biochemistry, Czech Academy of Sciences, 166 10 Prague 6, Czech Republic; 6 Laboratoire Chrono Environnement UMR CNRS6249, Université de Bourgogne Franche-Comté (UBFC), 16 route de Gray, 25030 Besançon, Cedex, France; 7 Receptor.AI Inc., 20-22 Wenlock Road, London, N1 7GU UK; 8 Department of Physical Chemistry, Faculty of Science, Palacký University Olomouc, 17. listopadu 12, 771 46 Olomouc, Czech Republic; 9 Department of Physics of Biological Systems, Institute of Physics of the National Academy of Sciences of Ukraine, Prospect Nauky 46, Kiev, 03028 Ukraine

**Keywords:** Fat, Lipids, Lipopolysaccharide, Taste buds, Inflammation

## Abstract

Inflammation is a mediator of a number of chronic pathologies. We synthesized the diethyl (9Z,12Z)-octadeca-9,12-dien-1-ylphosphonate, called NKS3, which decreased lipopolysaccharide (LPS)-induced mRNA upregulation of proinflammatory cytokines (IL-1β, IL-6 and TNF-α) not only in primary intraperitoneal and lung alveolar macrophages, but also in freshly isolated mice lung slices. The in-silico studies suggested that NKS3, being CD36 agonist, will bind to GPR120. Co-immunoprecipitation and proximity ligation assays demonstrated that NKS3 induced protein–protein interaction of CD36 with GPR120in RAW 264.7 macrophage cell line. Furthermore, NKS3, via GPR120, decreased LPS-induced activation of TAB1/TAK1/JNK pathway and the LPS-induced mRNA expression of inflammatory markers in RAW 264.7 cells. In the acute lung injury model, NKS3 decreased lung fibrosis and inflammatory cytokines (IL-1β, IL-6 and TNF-α) and nitric oxide (NO) production in broncho-alveolar lavage fluid. NKS3 exerted a protective effect on LPS-induced remodeling of kidney and liver, and reduced circulating IL-1β, IL-6 and TNF-α concentrations. In a septic shock model, NKS3 gavage decreased significantly the LPS-induced mortality in mice. In the last, NKS3 decreased neuroinflammation in diet-induced obese mice. Altogether, these results suggest that NKS3 is a novel anti-inflammatory agent that could be used, in the future, for the treatment of inflammation-associated pathologies.

**Supplementary Information:**

The online version contains supplementary material available at 10.1007/s00018-024-05207-1.

## Introduction

Inflammation is a normal response to infection of the host; however, a prolonged state of inflammation may cause a number of physiological complications. High-grade inflammation can lead to acute lung injury or septic shock, which is one of the leading causes of deaths worldwide. Chronic or low-grade inflammation may result into severe long-term consequences such as atherosclerosis, obesity, and diabetes [1].

Sepsis or septic shock is characterized by an overwhelming inflammatory response that leads to multiple organ failure, shock, and death. This is due to an overproduction of inflammatory cytokines, also referred to as “cytokine storm”. The cytokines like IL-6, IL-1β and TNF-α are among the major agents which are responsible for septic disease pathogenesis [2]. Septic shocks are mostly caused by the lipopolysaccharide (LPS), a major component of the cell wall of gram-negative bacteria [3]. Indeed, LPS is recognized, at the macrophage plasma membrane, by CD14 and TLR4, leading to the activation of the NF-kB pathway and the production of pro-inflammatory cytokines, such as IL-6, IL-1β and TNF-α.

A large number of clinical investigations have shown that fatty acids possess pro and/or anti-inflammatory properties. The properties of each lipid or its derivative are unique and have distinct effects on inflammation. Fatty acids can be oxidized and detected by inflammatory cells or incorporated into the inflammatory cell membrane [4]. Finally, fatty acids can directly act on inflammatory cells via fatty acid receptors like CD36 or GPR120, a member of G-protein coupled receptor (GPCR) family. The GPR120 has been shown to mediate a strong anti-inflammatory effect in macrophages by inhibiting NF-kB pathways [5]. Serval GPR120 agonists have been developed, but most of them are not suitable for in vivo studies due to poor pharmacokinetic properties [6].

Since the inflammatory state is a key factor involved in a number of pathological complications and GPR120 might exert anti-inflammatory properties, we have synthesized a novel chemical compound, i.e., diethyl (9Z,12Z)-octadeca-9,12-dien-1-ylphosphonate, termed as NKS3 hence after, on the chemical template of linoleic acid, a dietary long-chain fatty acid (see, our patent, US20210155640A1). In the present report, we describe the anti-inflammatory properties of this novel agent under different experimental conditions.

## Results

### NKS3 exerts anti-inflammatory action in primary cultured macrophages and lung slices

**Fig. 1:**
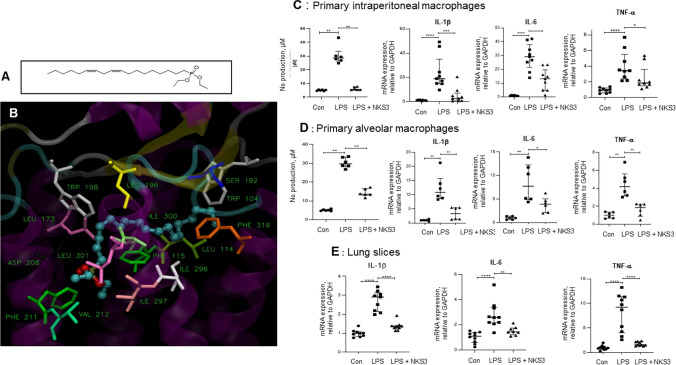
Newly synthesized NKS3 decreases LPS-induced inflammation in vitro and ex vivo. **A** Shows the chemical structure of diethyl-106 (9Z,12Z)-octadeca-9,12-dien-1-ylphosphonate, called hereafter as NKS3. The** B** shows the binding of NKS3 to GPR120 in ensemble docking simulations. GPR120 protein is shown in semi-transparent cartoon representation. The residues in contact with NKS3 (within 0.3 nm from any ligand atom) are shown as sticks, labelled and colored by residue number. Hydrogens and water molecules are omitted for clarity. **C–E** Show experiments conducted, respectively, on primary intraperitoneal macrophages, alveolar macrophages and freshly isolated lung slices from *C57BL/6* mice. The macrophages were first treated with NKS3 (50 µM for 2 h) and then LPS (10 ng/ml for 24 h). The NO production was determined in the supernatant by employing Griess reagent (**C**, **D**). The expression of mRNA encoding IL-1β, IL-6 and TNF-α were determined by RT-qPCR. Data are shown as median with interquartile range (n = 5 at least). In **E,** the slices were first treated with NKS3 (50 µM for 2 h) and then LPS (10 ng/ml for 24 h) and processed to study the expression of mRNA encoding IL-1β, IL-6 and TNF-α as mentioned in **C** and **D**. Data are shown as median with interquartile range (n = 5 at least). The control (Con) has been considered as 1 for RT-qPCR analyses. The nonparametric Mann–Whitney test was used for the statistical analyses (*p < 0.05; **p < 0.01; ***p < 0.001); ****p < 0.0001). *LPS* lipopolysaccharide, *Con* Control

We have synthesized diethyl (9Z,12Z)-octadeca-9,12-dien-1-ylphosphonate (Fig. 1A) as described elsewhere (patent US20210155640A1). We have reported previously that NKS3 is a CD36 agonist [7], however, the docking simulations suggest that this chemical compound will also bind GPR120. The distributions of the binding scores of NKS3 in ensemble docking simulations for CD36 and GPR120 were reported recently [7]. According to them the most probable binding score is − 5.8 kcal/mol for CD36 and − 6.75 kcal/mol for GPR120. This suggests that NKS3 is a dual binder for GPR120 and CD36 with better affinity to the former.

Our first and foremost aim was to assess the anti-inflammatory properties of this chemical compound. We employed three models: primary intraperitoneal and alveolar macrophages, and freshly isolated lung slices. Lipopolysaccharide (LPS) was used to induce inflammation, assessed by the expression of mRNA encoding inflammatory cytokines. Pretreatment with NKS3 decreased not only nitric oxide (NO) production in culture supernatants but also the mRNA expression of pro-inflammatory cytokines (IL-1β, IL-6 and TNF-α) in primary intraperitoneal and alveolar macrophages (Fig. 1C, D). Similarly, NKS3 decreased the LPS-induced mRNA expression of IL-1β, IL-6 and TNF-α in lung slices (Fig. 1E).

### NKS3 binding to CD36 promotes its interaction with GPR120 and GPR120-mediated downstream signaling in RAW 264.7 macrophage cell line

**Fig. 2:**
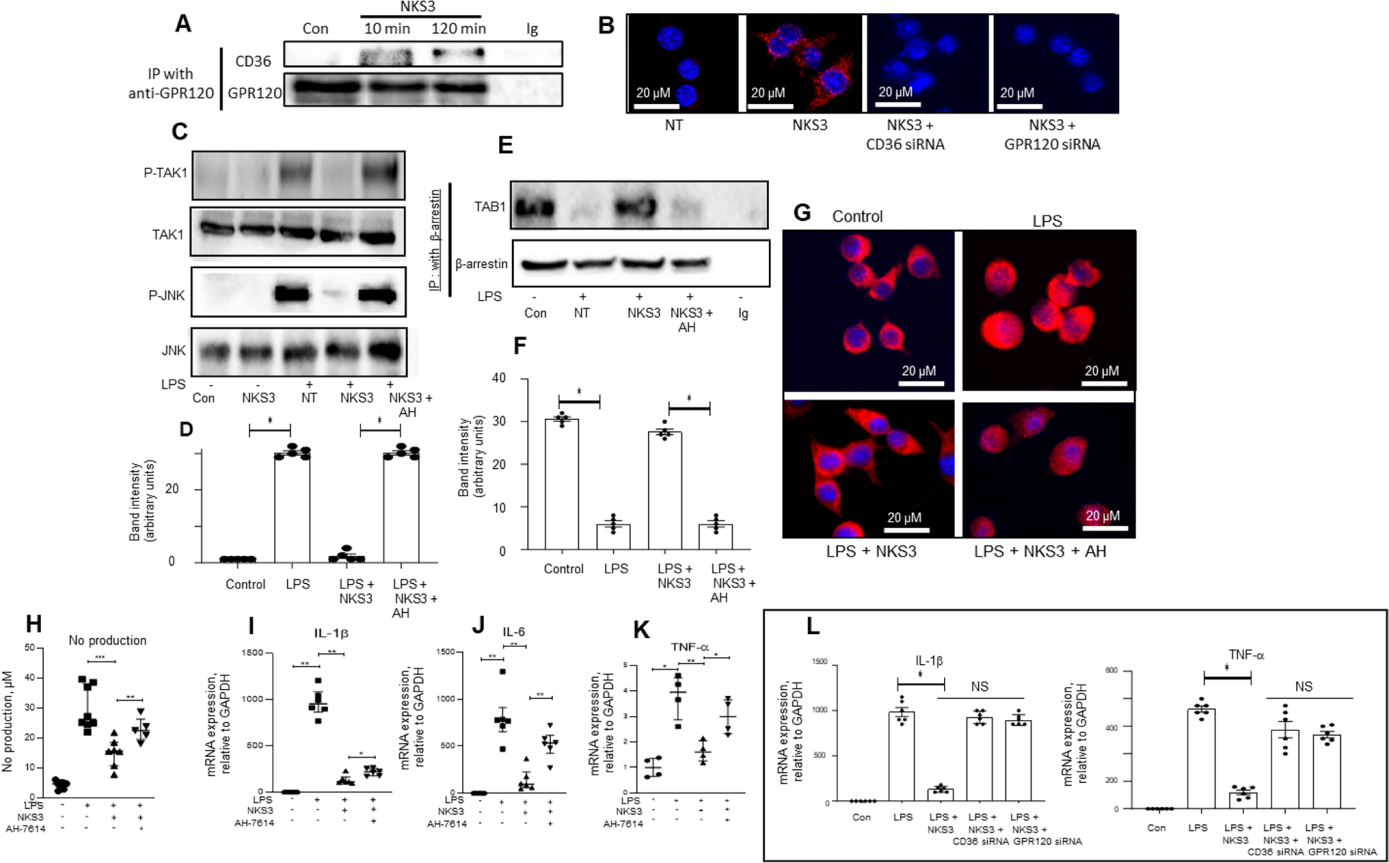
NKS3 binds to CD36 and triggers conformational protein–protein interaction with GPR120 and its downstream signaling in RAW 264.7 macrophage cell line. In **A**, the RAW 264.7 cells were treated with NKS3 (50 µM for 2 h) and then LPS (10 ng/ml for 24 h). The incubations were terminated by removing the supernatants and the lysates were used for GPR120 immuno-precipitation and detection of CD36 and GPR120 proteins by Western blotting. In **B**, the proximity ligation assay (PLA) was performed to demonstrate protein–protein (CD36 and GPR120) interaction/colocalization. The experiments were conducted on control (non-treated cells, NT) cells or the cells in which CD36 and GPR120 expression was knocked-down by siRNA. The cells were incubated with both anti-CD36 and anti-GPR120 antibodies and processed for PLA detection, after washing, in a Zeiss fluorescent microscope. Fluorescent dots represent cross-linked antibodies. In **C**, the lysates were used for TAK1, p-TAK1, JNK, and p-JNK expression by Western blotting (n = 3). In **E**, lysates were used for β-arrestin immuno-precipitation followed by Western blotting for immunodetection of phosphorylated TAB-1. **D**, **F** Show densitometric histograms whose values are derived from, respectively, **C** and **E** blots. The histograms represent mean band intensity means ± SD (n = 5, Western blots) of P-JNK (**C**) and TAB1 (**E**). **G** shows the nuclear localization of NF-kB in RAW 264.7 cells, untreated (control) or those treated with either of the followings: LPS or NKS3 or LPS + NKS3. In **H**, the routinely cultured RAW 264.7 cells were washed and treated with AH-7614 (25 µM for1h), NKS3 (50 µM for 2 h) and then LPS (10 ng/ml for 24 h). NO production was determined in the supernatant by employing Griess reagent. Data are shown as median with interquartile range (n = 5 at least). **I**–**K** show the expression of mRNA encoding IL-1β, IL-6 and TNF-α determined by RT-qPCR. Data are shown as median with interquartile range (n = 5 at least). The control (Con) has been considered as 1 for RT-qPCR analyses. **L** shows the mRNA expression encoding IL-1β and TNF-α in RAW 264.7 cells (as in **I**) that were either control (untreated; Con) or having silenced the expression of CD36 or GPR120 by siRNA technology (n = 6). *AH* AH-7614, *LPS* lipopolysaccharide, *NT* untreated cells, *Con* Control

In order to characterize the mechanism of action of NKS3 and to shed light at whether CD36 or GPR120 is responsible for its anti-inflammatory properties, we employed immunoprecipitation assays on RAW 264.7 macrophage cell line. The cells were activated by NKS3 and immunoprecipitated by anti-GPR120 antibodies, cell lysates were used for the detection of CD36 and GPR120 (Fig. 2A). We observed that immunoprecipitation with anti-GPR120 antibodies also revealed the presence of CD36, suggesting NKS3-induced coupling of CD36 and GPR120 in these assays.

It is noteworthy that transient, low-affinity interactions, which are common in GPCR signaling, are difficult to detect with co-immunoprecipitation. However, proximity ligation assay **(**PLA) offers the opportunity to visualize protein–protein interactions (at a distance of nearly 40 nm) and preserves low-affinity transient interactions or modifications. We observed that NKS3 in PLA experiments promoted the molecular interaction of CD36 and GPR120 which was abolished when the expression of CD36 or GPR120 was downregulated by siRNA technology (Fig. 2B and Suppl Fig. 1). This suggests that NKS3 induces conformational coupling of CD36 and GPR120 and the latter, being a GPCR, may mediate the NKS3-triggered downstream signaling.

To explore the downstream signaling mechanisms triggered by NKS3, the RAW 264.7 cells were pretreated with NKS3 for 2 h, followed by LPS stimulation that is known to induce the phosphorylation of TLR4 (Suppl Fig. 2). The NKS3 did not decrease the LPS-induced TLR-4 phosphorylation, suggesting that NKS3 would exert its action downstream to TLR-4. Interestingly, NKS3 strongly inhibited LPS-induced phosphorylation of JNK and TGF-beta activated kinase-1, TAK1 (Fig. 2C, D) without affecting cell viability (Suppl Fig. 3). The action of NKS3 was completely prevented by AH-7614, a GPR120 specific inhibitor, showing that NKS3 activity is GPR120-dependent. It has been previously reported that GPR120 may exert its anti-inflammatory properties through its association with β-arrestin2 and TAB1. Using immuno-precipitation, we demonstrate that NKS3 is able to increase β-arrestin/TAB1 complex (Fig. 2E, F). These data suggest that NKS3 exerts anti-inflammatory effects through GPR120 activation.

**Table 1:** The sequences of primers used in the study

GAPDH	Forward	5′-ACTCCACTCACGGCAAATTC-3′
	Reverse	5′-TCTCCATGGTGGTGAAGACA-3′
IL-1β	Forward	5′-TGTTCTTTGAAGTTGACGGACCC-3′
	Reverse	5′-TCATCTCGGAGCCTGTAGTGC-3′
IL-6	Forward	5′-CCGCTATGAAGTTCCTCTCTGC-3′
	Reverse	5′-ATCCTCTGTGAAGTCTCCTCTCC-3′
TNF-α	Forward	5′-CTCTTCTCATTCCTGCTTGTGG-3′
	Reverse	5′-AATCGGCTGACGGTGTGG-3′
Arginase-1	Forward	5′-AATGAAGAGCTGGCTGGTGT-3′
	Reverse	5′-CTGGTTGTCAGGGGAGTGTT-3′
iNOS	Forward	5′-GACATTACGACCCCTCCCAC-3′
	Reverse	5′-GCACATGCAAGGAAGGGAAC-3′

LPS has been reported, via TLR-4, to initiate translocation of the pro-inflammatory transcription factor NF-κB into the nucleus to trigger the transcription of target genes [8]. Figure 2G shows that LPS treatment promotes the translocation of NF-kB into the nucleus whereas the NKS3 treatment decreased its nuclear translocation. Conversely, the AH-7614, GPR120 inhibitor, blocked the action of NKS3 (Fig. 2G).

### NKS3 decreases inflammatory markers in LPS-treated RAW 264.7 cells via GPR120

We investigated the anti-inflammatory properties of NKS3 in RAW 264.7 cells. LPS alone triggered the NO production (Fig. 2H) and expression of mRNA encoding iNOS and arginase-1 (Suppl Fig. 4). LPS also upregulated expression of mRNA encoding IL-1β, IL-6 and TNF-α (Fig. 2I–K), whereas a pretreatment with NKS3 significantly decreased the LPS-induced NO production and cytokine mRNA expression (Fig. 2H, I–K). Interestingly, the GPR120 inhibitor, AH-7614, reversed the action of NKS3 on these pro-inflammatory parameters. To confirm that NKS3-mediated downstream signaling requires CD36-GPR120 physical interaction, we downregulated the expression of CD36 or GPR120 by siRNA which abrogated the inhibitory action of NKS3 on mRNA expression for IL-1β and TNF-α in these cells (Fig. 2L).

Our results suggest that NKS3 favors CD36-GPR120 interaction and triggers a downstream signaling cascade via GPR120. To further demonstrate the implication of GPR120 in the mechanism of action of NKS3, we conducted experiments on (1) STC-1 cells that express GPR120 receptors and, (2) on STC-1 cells, stably transfected with exogenous CD36 (STC-1/CD36). The STC-1 cells represent a good model to assess the action of a GPR120 agonist as they do not express CD36. Besides, they do not express GPR40. These cells are enteroendocrine cells that, upon activation via GPR120, release GLP-1 into extracellular environment. We also knocked-down the expression of GPR120 and CD36 in these cells, and observed that NKS3 induced the release of GLP-1 both in parental and STC-1/CD36 cells. GPR120-knock-down, as expected, abolished the action of NKS3 (Suppl Fig. 5). The CD36 siRNA in parental STC-1 cells failed to decrease GLP-1 secretion, but not in STC-1/CD36 cells wherein CD36 knock-down significantly curtailed the action of NKS3 on the release of STC-1, again suggesting that exogenous CD36 establishes an interaction with endogenous GPR120 (Suppl Fig. 5).

### NKS3 protects mice from acute lung injury

**Fig. 3:**
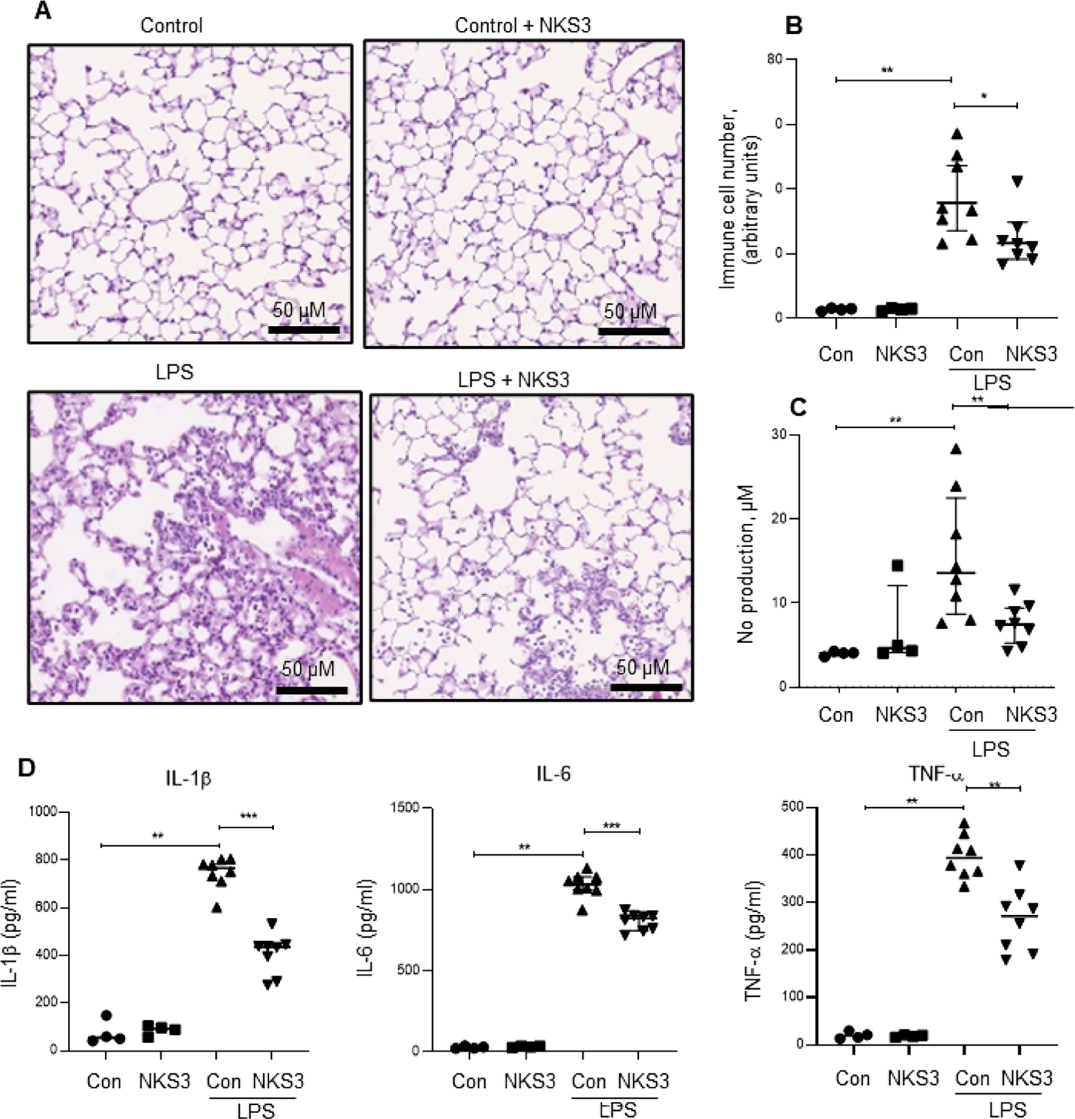
NKS3 reduces LPS-induced lung fibrosis and inflammation in broncho-alveolar lavage fluid (BALF) in mice. The *C57BL/6* mice were provided with NKS3 (at 50 µM) in their water bottles for 1 week. Later on, they were administered intra-tracheally with LPS (1.5 mg/kg) and scarified after 24 h of post-administration (n = 4–8 per group). After sacrifice, the lungs were fixed and subjected to paraffin embedding, and 5-μm sections were cut for staining with hematoxylin and eosin (× 20). The figure shows the representative images (**A**). **B** Shows the number of inflammatory cells in the lung, determined in histological slides using a manual counter. **C**, **D** Show, respectively, NO and cytokine production, determined by employing Griess reagent and ELISA respectively, in BALF. Data are shown as median with interquartile range. The nonparametric Mann–Whitney test was used for statistical analyses in **B**–**D** (*p < 0.05; **p < 0.01; ***p < 0.001). *AH* AH-7614, *BALF* bronchoalveolar lavage fluid

As our ex-vivo data shows that NKS3 was effective on lung tissues and alveolar macrophages, we decided to investigate the effect of NKS3 on a model of acute lung injury by intratracheal injection of LPS. The mice were supplemented in their water bottle (50 µM) by NKS3 for a week before the LPS injection and sacrificed 24 h later. The mice supplemented with NKS3 presented a decreased LPS-induced fibrosis (Fig. 3A) and inflammatory cells in their lung bronchoalveolar lavage fluid (BALF) (Fig. 3B). Similarly, NO production as well as IL-1β, IL-6 and TNF-α concentrations, in the presence of LPS, were also reduced by NKS3 in their BALF (Fig. 3C, D). Similar results were obtained on reduced mRNA expression of IL-1β, IL-6 and TNF-α in their lung (Suppl Fig. 6). NKS3 seems to be also able to reduce inflammation in ALI model.

### NKS3 protects mice against inflammation and septic death

**Fig. 4:**
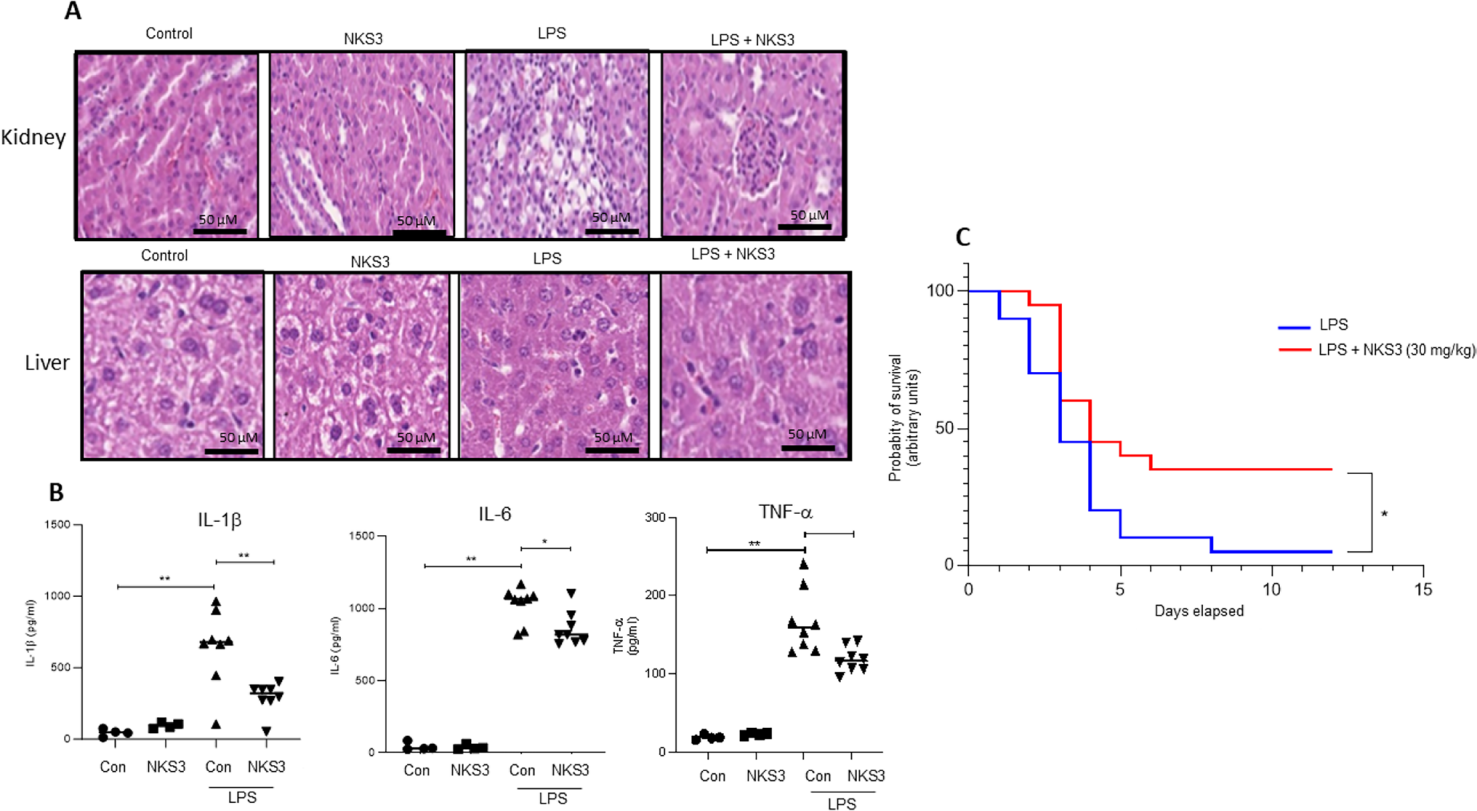
NKS3 decreases peripheral inflammation and enhances the survival rate of LPS-challenged mice. The *C57BL/6* mice were provided with NKS3 (at 50 µM) in their water bottles for 1 week. Later on, they were administered intraperitoneally with LPS (15 mg/kg) and scarified after 24 h of post-administration (**A**). After sacrifice, kidneys and liver samples were fixed and subjected to paraffin embedding, and 5-μm sections were cut for hematoxylin and eosin staining (**A**). **B** Shows IL-1β, TNF-α and IL-6 concentrations measured by ELISA in the sera of mice. Data are expressed as median with interquartile range. **C** Shows survival of mice after post-administration of LPS (n = 20). The mice were administered with NKS3 (30 mg/kg) orally 1 h prior to LPS injection (a lethal dose of 25 mg/kg). Mortality rate was recorded for 14 days after LPS injection. The nonparametric Mann-Whitney test was used for the statistical analyses in **B** (*p < 0.05; **p < 0.01). Mantel-Cox test was used for the statistical analyses in **C** (*p < 0.05)

Our in vitro and ex vivo results strongly suggest that NKS3 possess anti-inflammatory properties. We further investigated whether NKS3 treatment could be beneficial during a septic shock. Mice were first supplemented with NKS3 in their water bottle (50 µM) for one week prior to LPS injection and then sacrificed 24 h after injection. LPS-injected mice supplemented with NKS3 showed limited remodeling in their kidneys and liver (Fig. 4A). Moreover, the concentrations of IL-1β, IL-6 and TNF-α were reduced in their serum by NKS3 treatment (Fig. 4B). The LPS-induced changes in intestine length and spleen mass were also abolished by NKS3 treatment in mice (Suppl Fig. 7). To further investigate the effect of NKS3 on septic shock, we decided to inject mice with a lethal dose of LPS (25 mg/kg). The mice were administered with NKS3 orally (30 mg/kg) 1 h prior to LPS injection, and every 24 h onwards. Mortality rate was recorded for 14 days after LPS injection. We observed that NKS3 considerably extended survival of LPS-injected mice (Fig. 4C). Altogether, these data suggest that anti-inflammatory properties of NKS3 are effective on septic shock induced by LPS injection in mice.

### NKS3 protects mice against neuroinflammation in obese mice

**Fig. 5:**
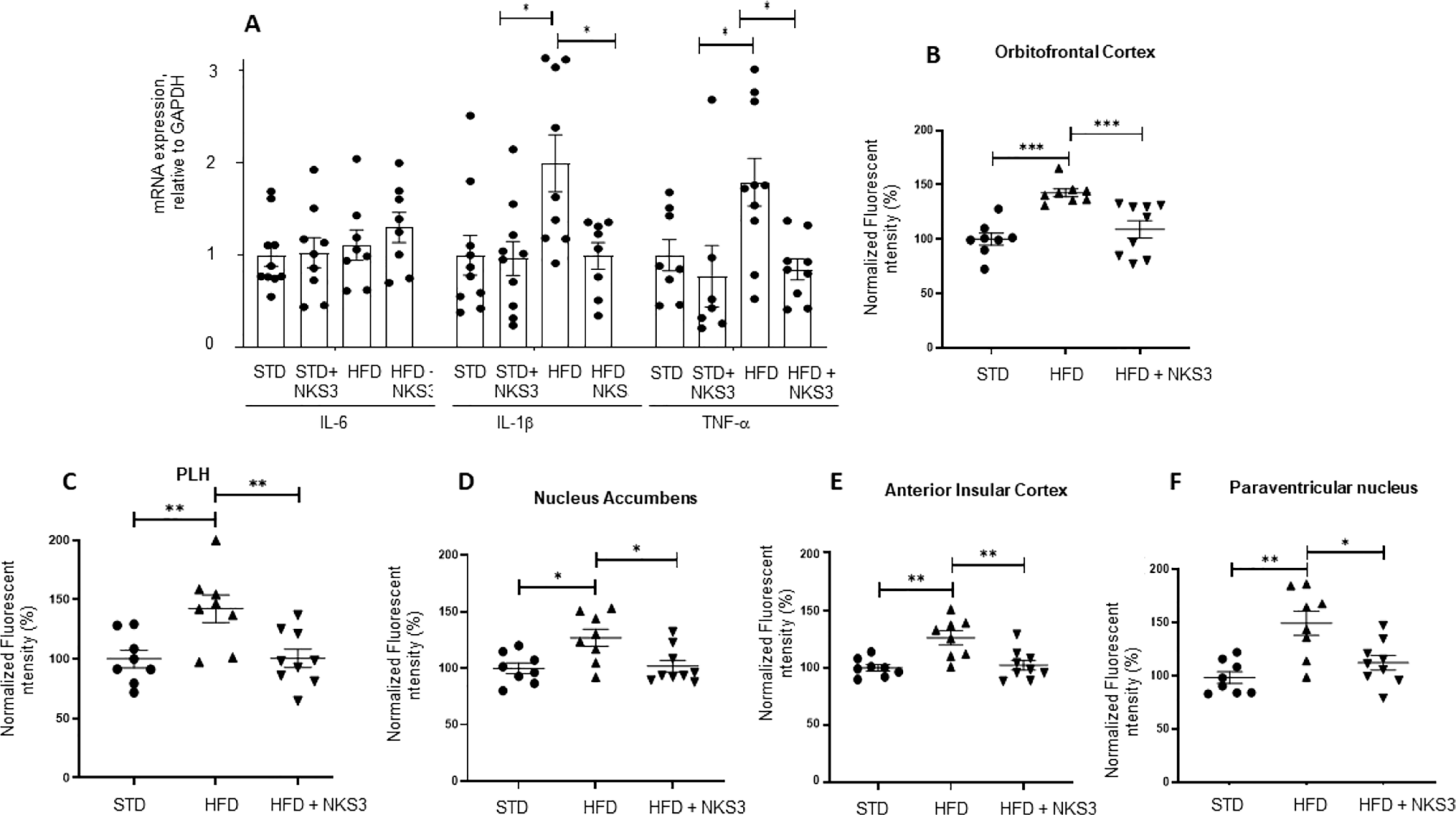
NKS3 also decreases pro-inflammatory cytokines and microglial cell neurotoxicity in the hypothalamus of diet-induced obese mice. The *C57BL/6* mice were fed a high-fat diet (HFD) for 17 weeks and were given or not ad libitum NKS3 (at 50 µM). The control animals received Arabic gum solution (0.3% w/v) for the same time period. After sacrifice, the hypothalamus was used for mRNA quantification for cytokines (**A**), and brains were fixed with a 4% paraformaldehyde solution (**B–F**) to study the expression of IBA1, a marker of microglia, in different brain regions. The nonparametric Mann–Whitney test was used for the statistical analyses (*p < 0.05; **p < 0.01). *PLH* penduncular part of the lateral hypothalamus

It has been well-demonstrated that obesity triggers low-grade inflammation not only in the peripheral tissues but also in the brain [9]. Obesity-induced neuroinflammation was first described in the hypothalamus, evidenced by the upregulation of JNK and NF-kB signaling and a reduced insulin and leptin profile caused by exposure to a high fat diet (HFD) [10]. Hypothalamic inflammation brings about alterations in neural projections, microgliosis and neuronal death [11]. During microgliosis, microglia’s activation may release pro-inflammatory cytokines such as IL-1β and TNF- α (9, 12). In the present study, the mice became obese through feeding them an HFD as described elsewhere [7]. We determined the expression of not only pro-inflammatory cytokines mRNA but also IBA-1, an indicator of microglial response, by immunofluorescence. The Fig. 5A shows that diet-induced obese mice exhibited high expression of IL-1β, IL-6 and TNF-α mRNA expression in the hypothalamic region and NKS3 exerted a significant inhibitory action on IL-1β and TNF- α mRNA levels. Furthermore, we observed that obese animals exhibited high expression of IBA-1 in the orbitofrontal cortex, the anterior insular cortex (Fig. 5B, E), some key hypothalamic regions, i.e., penduncular part of the lateral hypothalamus (PLH) and paraventricular nucleus (PVN), and in the nucleus accumbens, and that NKS3 significantly normalized this marker of gliosis (Fig. 5C, D, F).

## Discussion

A large number of anti-inflammatory drugs, currently in use, exhibit side effects with potential hazards in the long-run. The treatment of inflammation and, more precisely of chronic inflammation, would benefit from the development of new anti-inflammatory agents with lower side effects. Recent studies have shown that GPR120, a GPCR, may be an excellent target for the synthesis of novel anti-inflammatory drugs [12]. Therefore, the development of high-affinity molecules that could bind to GPR120 receptor and trigger anti-inflammatory effects would have a potential clinical application in the future.

Since polyunsaturated long-chain fatty acids like docosahexaenoic acid (DHA) and eicosapentaenoic acid (EPA) have been shown to exert anti-inflammatory properties via GPR120 activation in RAW 264.7 cells and primary intraperitoneal macrophages [5], in the present study, we have synthetized diethyl (9Z,12Z)-octadeca-9,12-dien-1-ylphosphonate based on the configuration of linoleic acid (patent US20210155640A1). We have recently shown that this agent, termed here as NKS3, binds to CD36; however, in silico studies indicate that this chemical compound will also bind to GPR120. Here, we were immediately interested in assessing its anti-inflammatory properties. We used lipopolysaccharide (LPS) to trigger inflammation in three models, i.e., primary intraperitoneal and alveolar macrophages, and freshly isolated lung slices. Nitric oxide (NO) has been reported to be a key player in endotoxin-induced tissue injury [13]. NKS3 decreased not only NO production but also mRNA expression of pro-inflammatory cytokines (IL-1β, IL-6 and TNF-α) in these experimental models.

These promising experiments encouraged us to elucidate the mechanism of action of NKS3 in a very stable macrophage model, i.e., RAW 264.7 cells. By immunoprecipitation and proximity ligation assay (PLA), we observed that NKS3 triggered the coupling of CD36 to GPR120. We also observed that CD36–GPR120 interaction was abolished by the knock-down (silencing) of either of the two proteins. It seems, therefore, that CD36 serves as a membrane co-receptor for GPR120. Indeed, CD36 has been shown to act as docking site for a number of proteins in lipid rafts [14]. Our results corroborate the findings of Hunag et al. [15] who have demonstrated, by employing PLA and anti-CD36 immuno-precipitates, that CD36 established a protein–protein interaction with CD9 in mouse peritoneal macrophages, upon activation by oxidized-LDL. Interestingly, the CD9 that recruits small GTP into plasma membrane domains [16], upon conformational binding to CD36, was responsible for the downstream signaling events as is the case of our study wherein GPR120, upon its recruitment/interaction, assures downstream signaling events (see hereafter). These observations partially corroborate our previous study in which we have demonstrated that CD36 is downregulated upon its activation, whereas concomitantly, GPR120 is upregulated into raft domains to promote cell signaling events in taste bud cells [17].

Furthermore, our data showed than upon NKS3 stimulation, GPR120 forms a complex with β-arrestin and TAB1. The formation of this complex results in the diminution of TAB1/TAK1 interaction and thus a decrease, by NKS3, in TAK1 phosphorylation in cells treated with LPS, leading to the inhibition of the NF-kB, a key regulator of inflammation and inflammatory diseases [18].

The short-lived free radical NO production by macrophages has been considered as an immediate defense against foreign agents [13]. As expected, NKS3 decreased, via GPR120 activation, NO production and expression of proinflammatory cytokines in these cells. RNA silencing further confirmed that the integrity of CD36-GPR120 association was primordial for the anti-inflammatory effects of NKS3 as the knock-down of CD36 or GPR120 abrogated the action of this compound on the expression of IL-1β and TNF-α mRNA. In order to validate the requirement of CD36-GPR120 interaction in NKS3-mediated cellular effects in another model, we used STC-1 cells that express functional GPR120 receptors as well as STC-1 cells stably expressing exogenous CD36 along with endogenous GPR120. The STC-1 cells are enteroendocrine cells that release GLP-1 upon GPR120 activation [19]. We observed that NKS3 triggered GLP-1 release in a GPR120-dependent manner in parental STC-1 cells; however, in cells co-expressing both CD36 and GPR120, the NKS3-induced GLP-1 secretion was blunted by the knock-down of either of the two proteins. An overall perusal of these observations confirms that the interaction of CD36-GPR120 plays an important role in the mechanism of action of NKS3 both in RAW 264.7 cells as well as in STC-1 cells. Moreover, NKS3 exerts its action via GPR120, but not via CD36, as regards downstream signaling cascade. In these experiments, we did not use sulfo-*N*-hydroxysuccinimidyl ester of oleate (SSO), a known CD36 inhibitor, as this agent establishes irreversible covalent binding with CD36 at the Lys^164^ residue, close to the hair-pin loop like structure of the CD36 [20], and consequently, may have blunted the CD36-GPR120 integration/association.

Hereafter, we used another model of inflammation, i.e., acute lung injury (ALI) or lung fibrosis. The ALI represents a syndrome of acute hypoxic respiratory failure resulting from direct and indirect injuries to the parenchyma of the lungs [21]. The mortality rate in patients with ALI or ALI-associated complications is ~ 40% [22]. The LPS induces symptoms in animal models that closely resemble to ALI in humans [23]. Our results clearly demonstrate a potent in vivo anti-inflammatory effect of NKS3 in the ALI model, where supplementation of NKS3 strongly reduced not only cytokine secretion in BALF, but also infiltration by inflammatory cells into the lung, as well as, limited lung fibrosis.

In the intensive care units, the septic shock represents one of the major challenges as it is responsible for life-threatening organ dysfunction due to dysregulated host responses to microbial infection [24]. In the septic choc, macrophage-secreted cytokines such as IL-1β and TNF- α have been proposed to be central mediators of its pathogenesis, responsible for the high mortality rate [25]. At first, we were interested in global inflammation and we studied, beside peripheral inflammation, the histological changes in liver and kidney. In fact, acute renal failure (ARF), reported in almost in 50% of patients with septic shock, plays a significant role in inducing organ damage [26]. We observed that NKS3 supplementation strongly reduced not only the concentrations of proinflammatory cytokines (IL-1β, IL-6 and TNF- α) in the blood, but also kidney and liver remodeling. Moreover, stronger concentration of NKS3 is able to increase survival rate of mice, administered with a lethal dose of LPS.

Beside peripheral inflammation, we were also interested in elucidating the anti-inflammatory action of NKS3 in neuroinflammation in diet-induced obese mice. Hence, we not only measured the mRNA expression of pro-inflammatory cytokines but also studied the gliosis which is the hallmark of the brain response to neuronal injury. In fact, microglia (macrophage-like cells in the brain) play a protective role in the brain. The Ionized Calcium Binding Adaptor molecule-1 (IBA1) is overexpressed in activated microglia following damage to the central nervous system. Our results on hypothalamic inflammation, marked with high expression of IL-1β and TNF- α mRNA, corroborate the interesting findings of Dorfman and Thaler [11] who have demonstrated that HFD-induced neuroinflammation is developed even after an acute (3 days) HFD feeding which induced the activation of microglia and the production of proinflammatory cytokines in the hypothalamus.

The penduncular part of the lateral hypothalamus (PLH) is very interesting area as it is involved in cortical activation and maintenance of behavioral arousal. The anterior insular cortex (ATC) has been considered as the gatekeeper of the executive control of the cognitive function [27], whereas orbitofrontal cortex (OFC) is known to be involved in inhibitory behavioral control. In fact, diet and stress induce architectural changes in OFC dendrites [28]. We observed that IBA1 expression was increased in PLH and OFC of obese mice. Our observations corroborate the findings of Veniaminova et al. [29] who have reported that a fat-rich western diet in mice induced the expression of IBA1 in the OFC. Décarie-Spain et al. [30] reported that the inflammation caused by feeding a HFD triggers pro-inflammatory signaling in the nucleus accumbens that might be at the origin of the emergence of anxio-depressive-like behaviors in male mice. The mechanisms through which NKS3 exerts regional anti-inflammatory actions in the brain are not yet fully understood. However, it is possible that NKS3-induced reduction in peripheral inflammation might be responsible for its anti-neuroinflammatory properties as suggested by Waise et al. [31] who have reported that HFD-induced upregulation of IBA1, IL-6 and TNF- α mRNA in hypothalamus might be result of the transfer of gut-derived signals to the hypothalamus via the nodose ganglion.

To sum up, we can state that NKS3 dramatically reduces pro-inflammatory signaling, including cytokine secretion in macrophage cell line, primary macrophages, peritoneal and alveolar macrophages, and increases the life expectancy in a model of acute endotoxemia in mice. NKS3 unveils novel opportunities for its application in combating chronic inflammation and related pathologies like atherosclerosis and rheumatoid arthritis.

## Methods

### Animal procedures

C57/bl6 J (WT) mice (Charles River, Saint Germain-sur-l’Arbresle, France) were housed in pathogen-free conditions. Food and water were provided ad libitum. The animals were treated according to the guidelines of the “Ministère de l’Enseignement Supérieur et de la Recherche” (Paris, France). All the experiments procedures were performed in strict accordance with the European Union regulations on animal research (Directive 2010/63/EU). All experiments were approved “APAFIS # 31988” by the “Comité d’Ethique de l’Expérimentation Animale du grand campus Dijon” (Bourgogne, France). The protocol for brain gliosis was also approved by the Ethical Committee (CEBEA-58).

The standard diet (A03) was purchased from Safe (France). It contained 5.1% of lipids as follows: monounsaturated fatty acids (palmitoleic acid, 1.05% and oleic acid, 21%) with low contents of saturated fatty acids (palmitic acid, 16.03% and stearic acid, 3.1%), and hight contents of polyunsaturated fatty acids (linoleic acid, 52% and alpha-linolenic acid, 5.9%). The HFD, used by us previously [7], was identical to standard diet in its composition except that it was enriched with palm oil that contained high contents of saturated fatty acids (palmitic acid, 43.5%; stearic acid, 4.3%), monounsaturated fatty acids (oleic acid, 39.8%) and low contents of polyunsaturated fatty acids (linoleic acid, 10%).

Intratracheal instillation of LPS at 1.5 mg/kg (Sigma) and intraperitoneal injection of LPS at 25 mg/kg were performed as previously described [32]. Mice were euthanized by abdominal aortic bleeding 24 h after administration. Bronchoalveolar lavage fluid (BALF) was collected as previously described [32].

### Synthesis of CD36 agonist and in silico modeling

Linoleic acid, a dietary long-chain fatty acid, was used as a precursor for the synthesis of diethyl (9Z,12Z)-octadeca-9,12-dien-1-ylphosphonate, i.e., C_22_H_43_PO_3_ (molecular weight, 386.56 Dalton). The details of the chemical synthesis can be seen in the published patent (US20210155640A1). Figure 1B was generated by VMD 1.9.3 (https://www.ks.uiuc.edu/Research/vmd/vmd-1.9.3/).

### Cell culture

The RAW 264.7 murine macrophage cells were routinely grown as monolayers with 5% CO_2_ and 95% air at 37 °C in RPMI medium (Lonza, Paris, France), supplemented with 10% of Fetal Bovine Serum (FBS, Lonza). Cells were seeded at 40% confluence one day prior to starting the treatment and then stimulated with LPS (Sigma) in complete medium at 10 ng/ml, with or without AH-7614 (25 µM), containing or not NKS3 (50 µM).

### Isolation of mice intraperitoneal and alveolar macrophages

Peritoneal macrophages were isolated from 8-weeks old male C57BL/6 mice, as described by Lu et al. [33]. A volume of 5 ml of PBS-EDTA was injected into the peritoneal cavity, after 10–15 s of abdomen massage, a syringe with 20 G needle was inserted into the abdomen to withdraw the fluid from peritoneum. After centrifugation (250 × g at 4 °C for 10 min), the cell pellet was suspended in antibiotic-free RPMI-1640 medium with 10% FBS. The cells (2 10^6^/well) were seeded onto 6-well cell culture dishes and cultured for 8 h. This allows the peritoneal macrophages to adhere to the plastic surface and non-adherent cells were removed by changing the media. Cells were used for the stimulation or not by LPS in order to conduct a western blot or a quantitative real-time PCR assay.

To purify the alveolar macrophages, we used 8-weeks old male C57BL/6 J mouse. The mice were anesthetized and BALF was recovered after the injection of 10 ml ice-cold PBS into the right ventricle [33]. After isolating the cells, by centrifuging at 250 × *g* at 4 °C for 10 min, the cells were suspended in 1 ml of culture medium [DMEM supplemented with 10% FBS, 20% L-929 culture supernatant (as a source of M-CSF, 100 U/mL final concentration), 1 mM sodium pyruvate, 10 mM HEPES, and 1 × penicillin/streptomycin (optional)].

### Precision cut lung slice preparation

3D-lung tissue slices were generated as previously described [32] and cultured for 72 h in DMEM medium containing 10% serum, 1% l-glutamine and 1% penicillin/streptomycin. Three slices were pooled for each mRNA extraction.

### Culture of STC-1 cells

The STC-1 cells were routinely cultured in DMEM medium with 20% FBS. STC-1 cells expressing human CD36 gene were generated by electroporation (Nucleofector Kit V, Lonza, Germany) and provided by Dr Nada Abumrad (Washington University, USA).

### Cells treatment

When it is not indicated in the legends, the cells were first preincubated with 50 µM NKS3 for 2 h and then with 10 ng/mL lipopolysaccharides (LPS).

### RT-qPCR analysis

Total mRNA from total lungs, RAW 264.7 cells, primary peritoneal macrophages, primary alveolar macrophages and 3D-lung tissue slices was extracted using TRIzol (Invitrogen, Carlsbad, USA). Reverse transcription was performed on total mRNA using the M-MLV kit (Promega, Charbonnieres, France). Quantitative RT-PCR (ViiA 7 Real-Time PCR System, ThermoFischer Scientific, Waktham, USA) was performed on the obtained cDNA using SYBR green master mix (ThermoFisher Scientific) using the mouse primers shown in the Table-I.

### Western blotting

Thirty µg of proteins were loaded onto 10% polyacrylamide gels. The PVDF membranes were incubated overnight at 4 °C with specific antibodies, anti-CD36 (rabbit Ab, NB400, Novus Biologicals), anti-GPR120 (rabbit Ab, NBP1-00858, Novus Biologicals), anti-β-arrestin2 (rabbit Ab, NB300-587, Novus Biologicals), anti-TAB1 (rabbit Ab, NBP1-76595, Novus Biologicals), anti-TAK1 (rabbit Ab, NBP1-76441, Novus Biologicals), anti-P-TAK1 (rabbit Ab, NBP3-21597, Novus Biologicals), anti-P-JNK (rabbit Ab, 9251, Cell Signaling), anti-JNK (rabbit Ab, 9252, Cell Signaling), anti-TLR4 rabbit Ab, 1005658) at a dilution of 1:500. Anti-β-Actin antibody (mouse Ab, 8H10D10, Cell Signaling) was used as a loading control. HRP-conjugated antibodies were used as secondary antibodies. Immunoblots were then incubated with goat anti-rabbit or goat anti-mouse antibodies (Cell Signaling) conjugated to horseradish peroxidase and developed by using the ECL method and reagents according to the manufacturer’s protocol (PerkinElmer, Waltham, MA, USA).

### Proximity ligation assay

RAW 264.7 cells were seeded on coverslips and cultured in RPMI-1640 medium supplemented with 10% FCS. The attached cells were fixed in 4% paraformaldehyde (PFA) at 37 °C for 15 min and permeabilized with 0.2% Triton in PBS at room temperature for another 15 min. Duolink^®^ In Situ Red Starter Kit Mouse (DUO92102/Rabbit DUO92104, Sigma) was used according to manufacturer’s instructions. Anti-CD36 mouse monoclonal antibody (NB400, Novus biologicals) and anti-GPR120 rabbit polyclonal antibody (NBP1-00858, Novus biologicals) were used as primary antibodies. Amplification and detection of bound PLA^®^ probes was performed with Duolink^®^ in situ detection red reagents (DUO92008, Sigma). Cells were counterstained with DAPI to detect nuclei. Distinct spots representing single-molecule protein interaction events were visualized using a confocal microscope (Zeiss LSM800) and analyzed using ImageJ software.

### Nuclear localization of NF-kB

RAW 264.7 cells were grown on sterile microscope slides, and treated with NKS3 (50 µM for 2 h) and then LPS (10 ng/ml for 24 h). At the end of the incubation, cells were washed with PBS and slides were fixed in 95% ethanol and rehydrated in 0.1 M PBS (pH 7.4). Slides were blocked in PBS containing 5% fetal calf serum and 0.2% Triton-X100 for 30 min at room temperature before overnight incubation at 4 °C with NF-κB p65 antibody (Cell Signalling, D14E12) (1/100 dilution). After washing, slides were incubated for 2 h at room temperature with rhodamine-conjugated secondary antibodies. Staining specificity was assessed by treating slides in the absence of primary antibodies. After three washings with PBS, a drop of ProLong Antifade reagents containing DAPI (Life technologies) was added onto the slide for the analysis under fluorescent microscope (Zeiss Axioskop).

### IL-1β, TNF-α and IL-6 quantification by ELISA

IL-1β, TNFα and IL-6 concentrations from BALF or plasma were determined using ELISA (Biolegend), according to the recommendations of the manufacturer.

### NO assay

The RAW 264.7 cells or primary (intraperitoneal or alveolar) macrophages were cultured in 96 well plates at density of 5 × 10^5^ cells/ml. The cells were treated as follows: normal medium or control, medium supplemented with 10 ng/ml LPS) containing or not the NKS3 or other agents [13]. After a 24 h of incubation at 37 °C and 5% CO_2_, culture supernatants were collected for NO (and cytokine) determination, using the Griess reaction in 96-well plates (Nunc, Denmark). The supernatant was mixed with equal volume of Griess reagent made up of 1% sulphanilamide (Sigma), 0.01% naphthy ethylenediamine dihydrochloride (Sigma), and 2.5% phosphoric acid. The color developed after a 15 min an incubation was measured at 540 nm using a microplate reader (Thermo electron). The concentration of NO was determined from a standard curve generated using sodium nitrite (100–1.56 μM).

### siRNA knock-down of CD36 and GPR120

To address the implication of GPR120 in protein–protein coupling, we used CD36 and GPR120 siRNA as per our protocol mentioned elsewhere [17]. siRNA silencing of CD36 and GPR120 in STC-1 cells and RAW 264.7 macrophage cells was brought about by transfecting with siRNA for human and mouse, respectively. The ONTarget plus Smart pool (25 nM) or nontargeting siRNA as a control (Dharmacon, Pittsburgh, PA) were emplolyed as per DharmaFECT protocol (Dharmacon). After 24 h of transfection, the cells were further cultured for 48 h in fresh medium and used for the experiments.

### Measurement of GLP-1 release from STC-1 cells

The STC-1 cells were cultured in DMEM medium with 20% FBS. The GLP-1 concentrations in the supernatants were determined by ELISA (Millipore) according to the protocol furnished with the kit. The STC-1 (parental and CD36-expressing) cells were incubated at 37 °C in an oxygenized medium containing NKS3. After 1 h of incubation, the supernatant was collected, and the active GLP-1 release was measured by ELISA. Dipeptidyl peptidase 4 (DPP4) inhibitor (0.1%; Millipore) was added to the medium to prevent GLP-1 degradation [17].

### Diet-induced obesity and IBA1 brain detection by immunofluorescence

Thirty-two C57BL/6J male mice aged of 8 weeks were maintained on a standard laboratory diet (STD) or a high-fat diet (HFD) as per our previously established protocol [7]. After 12 weeks of differential regime, the STD and HFD fed mice (n = 10 per group) were treated with vehicle (VEH; 0.3% Arabic gum solution; G9752, Sigma-Aldrich, France) for 5 weeks. The remaining HFD fed individuals were treated with a NKS3 solution (50 µM in VEH). Treatments were prepared every second day and animals received the respective solutions in water bottles. After 5 weeks of NKS3 treatment, animals were sacrificed for evaluation of the neuroinflammation by IBA-1 immunofluorescence. The procedures were similar to those previously described by our team [34, 35]. Briefly, after an intraperitoneal injection of pentobarbital (55 mg/kg, Exagon^®^, Med’Vet, France), mice were transcardially perfused with 0.9% NaCl, followed by ice cold 4% PFA fixation in 0.1 M phosphate buffer (PB, pH 7.4). Brains were extracted and post-fixed overnight in the same fixative at 4 °C and cryoprotected by immersion in a 15% sucrose solution (D[+)-Saccharose in 0.1 M PBS for 24 h at 4 °C. After freezing by immersion in isopentane, brains were sliced in coronal 30-µm-thick serial sections and stored at − 40 °C in a cryoprotective solution (1:1:2 glycerol/ethylene glycol/PBS).

IBA-1 expression was evaluated in hypothalamic, cortical and subcortical brain regions. For that, the following tissue sections were selected: nucleus accumbens (NAc; core-NAcC, and shell-NAcS) at (1.94–1.70) anterior to Bregma (aB); anterior insular cortex at (1.10–0.98) aB; paraventricular nucleus of the hypothalamus (PVN) at (0.26–0.02) aB and penduncular part of the lateral hypothalamus (PLH) at (1.06–1.34) posterior to Bregma (pB) according to Franklin and Praxinos (2008). After washing, floating sections were incubated with the primary antibody (1:2000; ab178846, rabbit anti-IBA1, Abcam) during 24 h at 4 °C. Tissues were then incubated with the secondary antibody (1:1000; A10520, goat anti-rabbit IgG Cyanine 3, Invitrogen) during 2 h at room temperature. Finally, sections were washed, mounted on gelatin coated slides and cover slipped with mounting medium (40% PB, 60% glycerol; Roth^*®*^, Karlsruhe, Germany). Photomicrographs of brain structures were acquired using 10 × objectives in Olympus microscope B × 51, equipped with a camera Olympus DP50. ImageJ software was used to quantify IBA1 related fluorescence over the regions of interest.

### Statistical analysis

Comparisons between different groups were performed using the non-parametric Mann–Whitney test and Prism 8 software (GraphPad, San Diego, CA, USA). Survival curves were evaluated with Mantel Cox test. *P* values below 0.05 were considered statistically significant. All results are representative of at least 3 different experiments.

### Supplementary Information

Below is the link to the electronic supplementary material.

Supplementary file1 (DOCX 360 KB)

## Data Availability

The data will be provided with upon a reasonable request.
