# Supplementary material for: A novel fatty acid analogue triggers CD36–GPR120 interaction and exerts anti-inflammatory action in endotoxemia
Source: Cell Mol Life Sci. 2024 Apr 10;81(1):176. doi: 10.1007/s00018-024-05207-1 (PMC11006773; doi:10.1007/s00018-024-05207-1)
Supplement: Supplementary file 1 — Supplementary file1 (DOCX 360 KB) [file 18_2024_5207_MOESM1_ESM.docx]

**Supplemental Figures**

**
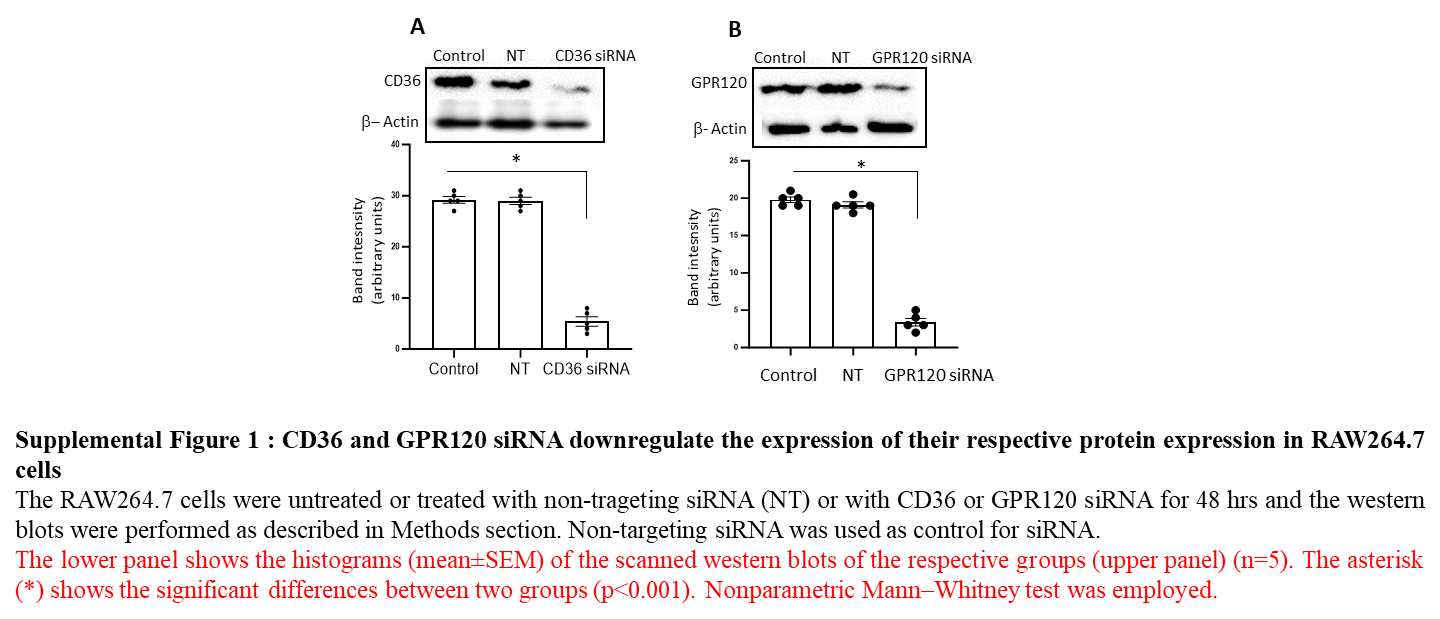
**

**Supplemental Figure 1 : CD36 and GPR120 siRNA downregulate the expression of their respective protein expression in RAW264.7 cells**

The RAW264.7 cells were untreated or treated with non-trageting siRNA (NT) or with CD36 or GPR120 siRNA for 48 hrs and the western blots were performed as described in Methods section. Non-targeting siRNA was used as control for siRNA.

The lower panel shows the histograms (mean±SEM) of the scanned western blots of the respective groups (upper panel) (n=5). The asterisk (*) shows the significant differences between two groups (p<0.001). Nonparametric Mann–Whitney test was employed.

**
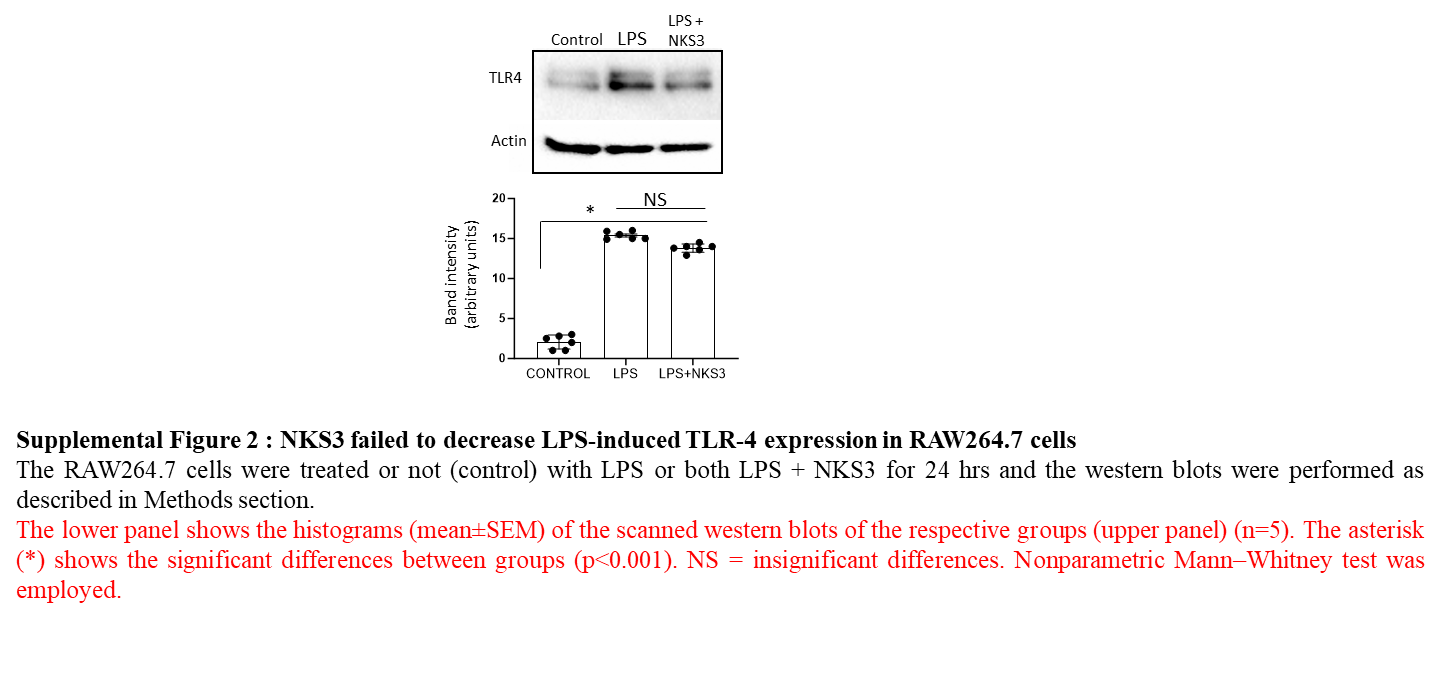
**

**Supplemental Figure 2: NKS3 failed to decrease LPS-induced TLR-4 expression in RAW264.7 cells**

The RAW264.7 cells were treated or not (control) with LPS or both LPS + NKS3 for 24 hrs and the western blots were performed as described in Methods section.

The lower panel shows the histograms (mean±SEM) of the scanned western blots of the respective groups (upper panel) (n=5). The asterisk (*) shows the significant differences between groups (p<0.001). NS = insignificant differences. Nonparametric Mann–Whitney test was employed.

**
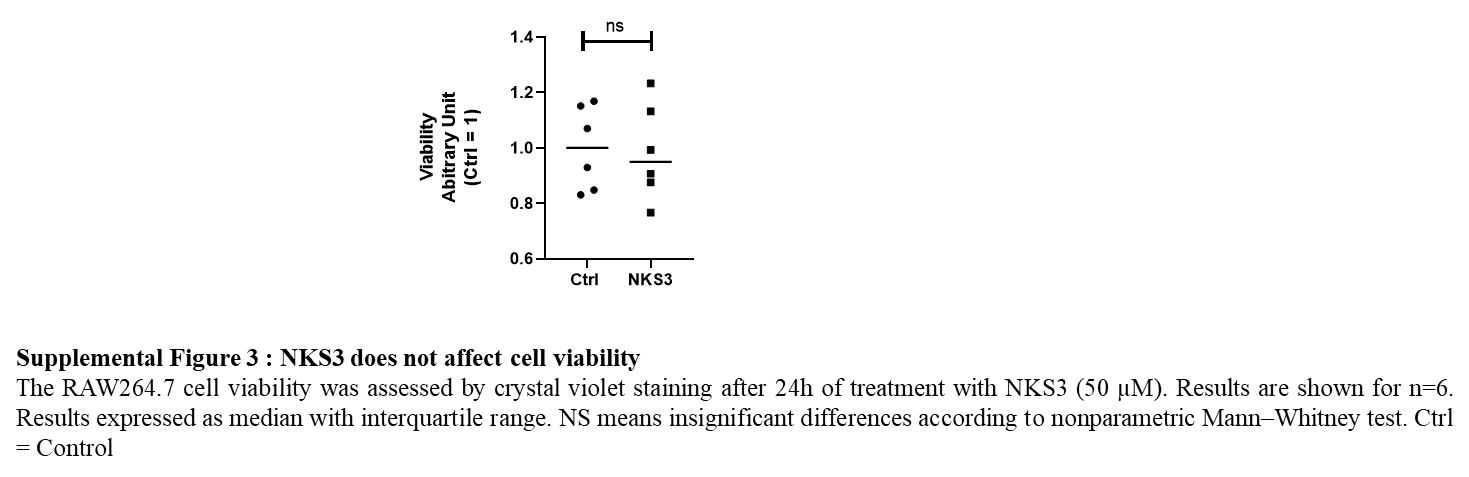
**

**
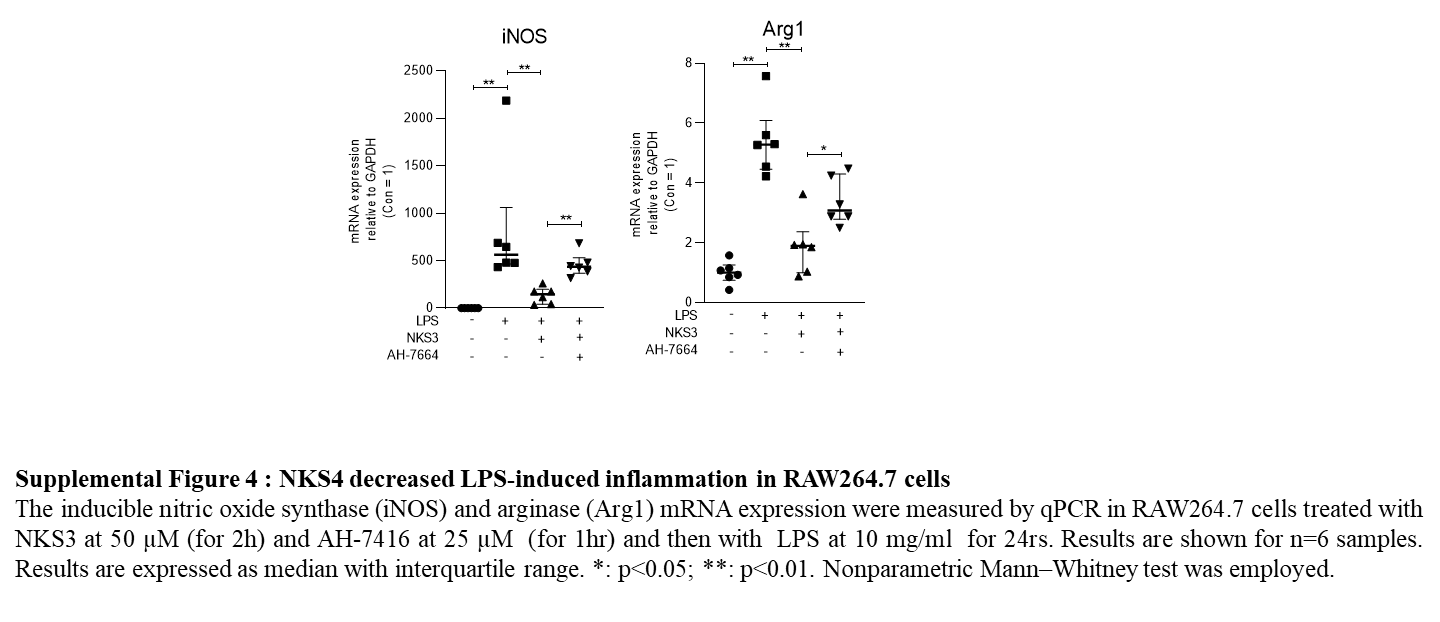
**

**
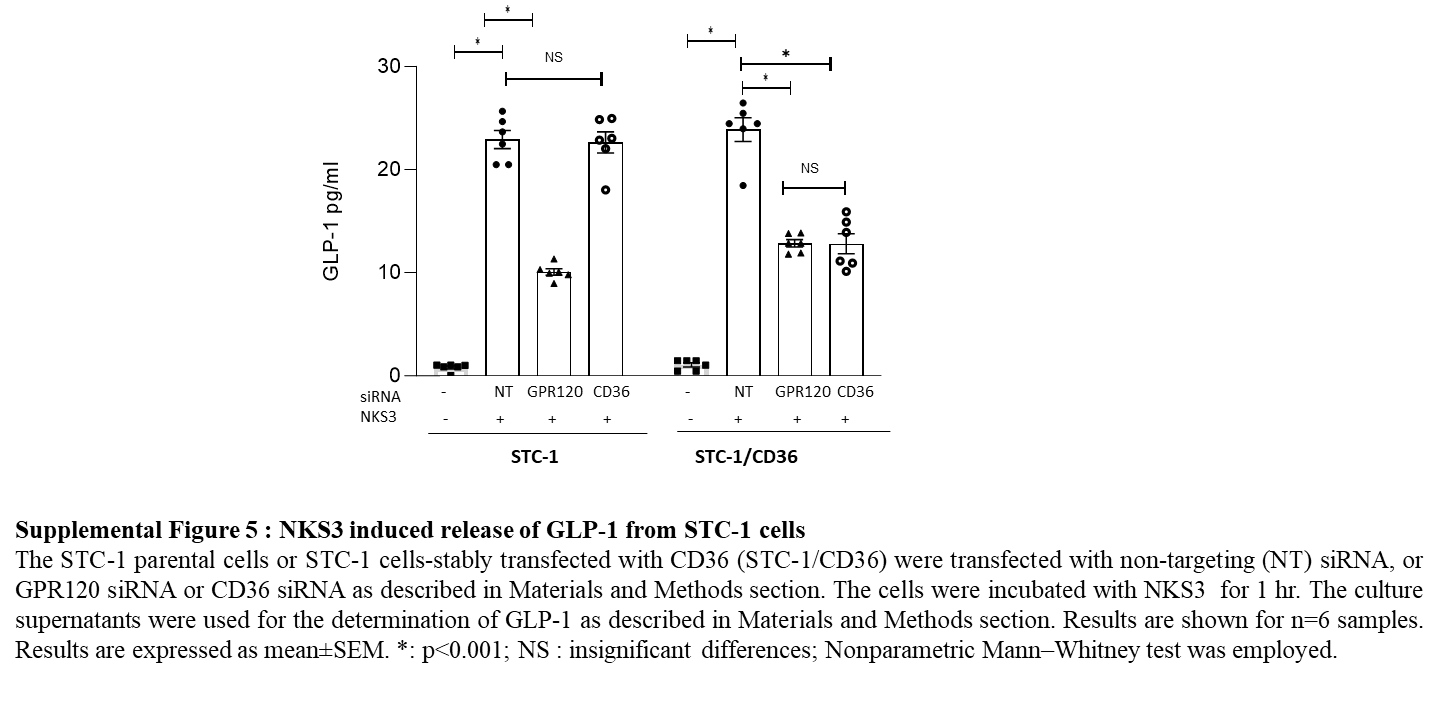
**

**
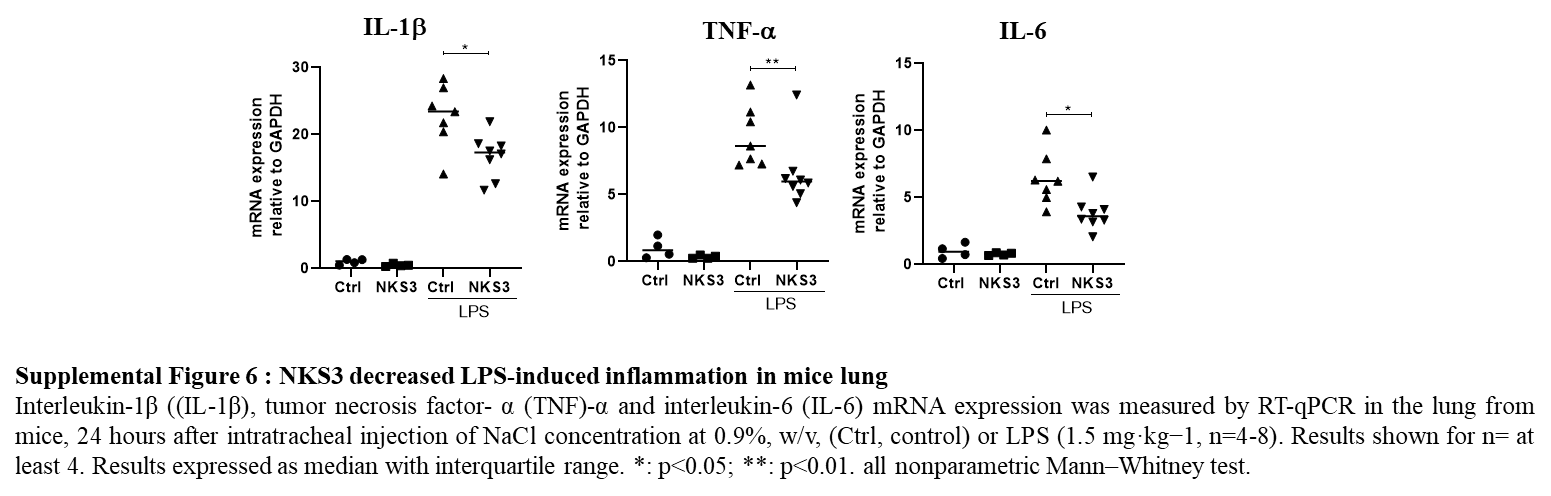
**

**
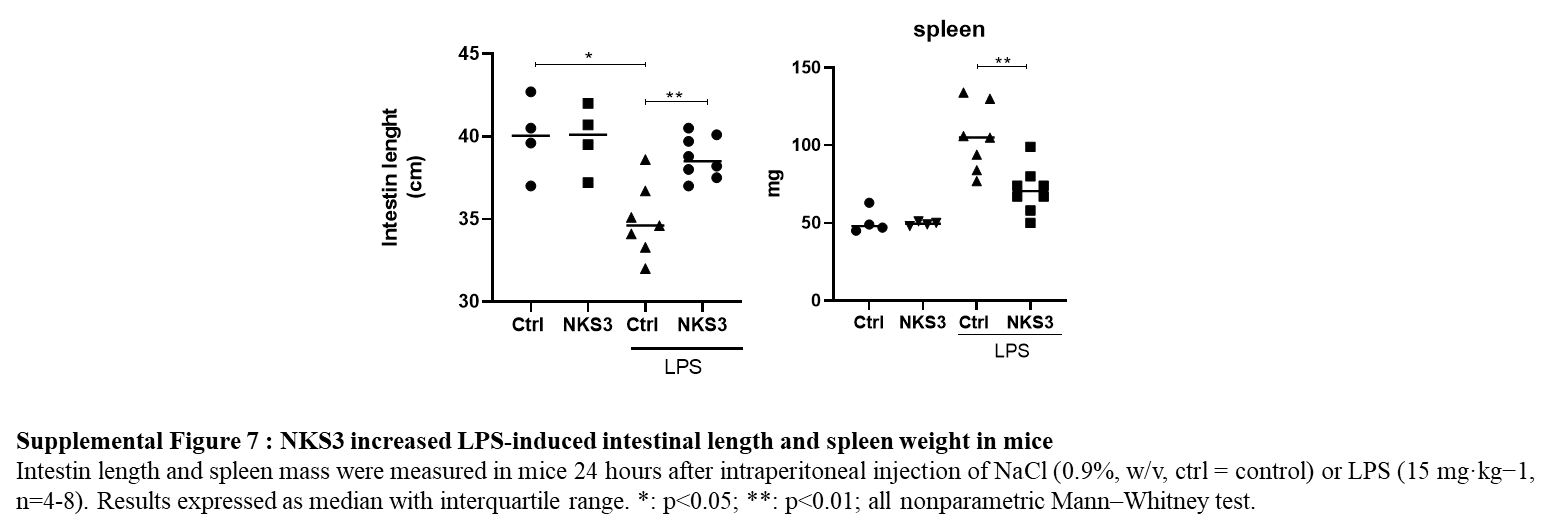
**
